# Metagenome Analysis Reveals Changes in Gut Microbial Antibiotic Resistance Genes and Virulence Factors in Reintroduced Giant Pandas

**DOI:** 10.3390/microorganisms13071616

**Published:** 2025-07-09

**Authors:** Wanju Feng, Chenyi Gao, Xinyuan Cui, Bing Yang, Ke He, Qiuyu Huang, Xinru Yang, Kaizhi Wen, Jiadong Xie, Zhisong Yang, Lifeng Zhu

**Affiliations:** 1 Sichuan Academy of Giant Panda, Chengdu 610081, China; 19828320401@163.com (W.F.); xinyuan890@163.com (B.Y.); heke0611@163.com (K.H.); 2 School of Medicine, Nanjing University of Chinese Medicine, Nanjing 210023, China; 17826720536@163.com (C.G.); cuixinyuanwj@163.com (X.C.); hqy202016@163.com (Q.H.); yxr1457448137@163.com (X.Y.); 13290558270wkz@sina.com (K.W.); 3 School of Artificial Intelligence and Information Technology, Nanjing University of Chinese Medicine, Nanjing 210023, China; xiejdm@njucm.edu.cn; 4 Jiangsu Province Engineering Research Center of TCM Intelligence Health Service, Nanjing 210023, China; 5 Key Laboratory of Drug Target and Drug for Degenerative Disease, Nanjing University of Chinese Medicine, Nanjing 210023, China

**Keywords:** giant pandas, metagenome, antibiotic resistance gene (ARG), virulence factor (VF)

## Abstract

Antibiotic resistance has emerged as a critical global public health challenge. In this study, we employed metagenomic sequencing to analyze fecal samples from giant pandas (*Ailuropoda melanoleuca*) across three distinct stages—semi-wild, released, and wild populations—to investigate shifts in antibiotic resistance genes (ARGs) and virulence factors (VFs) during the reintroduction process. Our findings revealed significant variations in the composition of ARG and VF across different stages, with released and wild giant pandas exhibiting similar ARG and VF profiles. Further analyses identified that the increased abundance of ARGs and VFs in both released and wild individuals compared to semi-wild individuals was mainly from *Pseudomonas*. We hypothesized that the same geographic environment in which ARGs and VFs are transmitted between a host and the environment via mobile genetic elements (MGEs) may be responsible for the similar structure of ARGs and VFs in released and wild giant pandas. Additionally, diet may modulate the gut microbial community, thereby influencing the distributions of ARG and VF. This study elucidated the impact of geographic and dietary factors on ARGs and VFs dynamics in giant pandas, offering valuable insights for mitigating antibiotic resistance and virulence gene dissemination.

## 1. Introduction

Giant pandas (*Ailuropoda melanoleuca*) are a globally endangered species [1], and their population decline is primarily driven by habitat loss and fragmentation [2]. Beyond these environmental pressures, intestinal infections present a critical threat to giant pandas’ survival. Several opportunistic enteropathogens in giant pandas, including *Escherichia coli*, *Klebsiella pneumoniae*, *Campylobacter jejuni*, *Pseudomonas aeruginosa*, *β-hemolytic streptococci*, and *Clostridium welchii*, were considered to probably cause intestinal diseases in giant pandas [3]. Antibiotics are commonly administered to treat giant pandas’ infections. However, the pervasive use of these pharmaceuticals has precipitated the emergence of multidrug-resistant (MDR) bacterial strains. Notably, some studies have isolated multidrug-resistant pathogens from giant pandas [4,5,6], including *Klebsiella pneumoniae*, *Escherichia coli*, and *Enterococcus faecium*, which indicate a growing threat to the survival and health of giant pandas.

Antibiotic resistance has emerged as a critical global public health challenge, requiring urgent action under the “One Health” framework. The “One Health” framework emphasizes an integrated approach to combat antibiotic resistance by recognizing the interconnected roles of animal health, human health, and environmental ecosystems, avoiding the cross-species transmission of antibiotic resistance genes (ARGs) across the animal–environment–human interface [7,8]. ARGs are genetic elements within microbial genomes that mediate resistance to antibiotics. ARGs are considered to be the main driver of bacterial resistance [9], which can facilitate the spread of resistance genes in the environment via mobile genetic elements (MGEs), resulting in the accumulation of resistance genes [10]. Notably, wildlife is recognized as a sentinel victim of environmental contamination and antibiotic misuse in human/livestock populations [11]. This underscores the critical need for systematic surveillance of ARGs in wildlife ecosystems. Concurrently, virulence factors (VFs) are considered to exacerbate pathogenic infections by enhancing microbial colonization, immune evasion, and host immunosuppression [12]. It has been shown that the metalloprotease LasB, a key virulence factor of *Pseudomonas aeruginosa*, can promote bacterial colonization of the host, disrupt host immunity, and cause cytotoxicity [13]. In addition, *Escherichia coli* can utilize the KpsM virulence factor to form a capsule, mimicking host-surface structures to evade hepatic macrophage phagocytosis [14]. It is generally believed that bacterial resistance and high virulence cannot coexist. A recent study has documented the emergence of *Klebsiella pneumoniae* strains exhibiting concurrent multidrug resistance and hypervirulence phenotypes [15]. In summary, both ARGs and VFs can adversely affect the survival and health of wildlife. These findings underscore the critical need for systematic surveillance of ARGs and VFs. Investigating the dynamic changes in ARGs and VFs during giant panda reintroduction programs could provide important theoretical guidance on the rational use of antibiotics to treat infectious diseases in giant pandas.

Reintroduction initiatives represent a pivotal strategy for the conservation of endangered species, with notable achievements in the protection of giant pandas. It has been shown that the wildness of the gut microbiomes was restored (high proportion of *Pseudomonas*), and the function was enriched in amino acid metabolic activity during the translocated giant pandas’ release into the wild, which may demonstrate the adaptation of giant pandas to their environment [16]. In addition, Huang et al. revealed that the gut phenotype can be used as a monitoring indicator for reintroduction. The reintroduced giant pandas gradually developed a *Pseudomonas* enterotype similar to the wild giant pandas, which showed the enriched cellulose-degrading enzymes, helping them adapt to the highly fibrous diet in the wild [17]. The activity rhythms and monthly activity rates have also been found to show similarity between the reintroduced giant pandas and wild giant pandas [18]. Reintroduced giant pandas have exhibited adaptive responses to the wild environment, showing progressive convergence in gut microbial composition, function, and activity patterns toward wild giant pandas. However, the dynamics of ARGs and VFs in giant panda populations during reintroduction remain poorly characterized.

Gastrointestinal diseases caused by pathogens remain a leading cause of mortality in giant pandas [19]. The ARGs and VFs carried by pathogens can pose significant challenges for the clinical management of infectious diseases. Many studies have characterized the ARG and VF profiles of the bacteria isolated from giant pandas, with significant reservoirs of ARGs and VFs identified in *Escherichia coli* [5], *Enterococcus faecium* [6], and *Klebsiella pneumoniae* [20]. Additionally, the possible drivers shaping the different ARGs and VFs in giant pandas have been explored. A comparative analysis of ARGs and VFs between captive and wild giant pandas revealed distinct profiles, with significantly higher abundances observed in captive individuals. This disparity likely stemmed from an artificially polluted captive environment [21]. Furthermore, geographic variation, dietary composition, and host age have been implicated as determinants shaping ARG and VF distributions [3,22,23,24]. Current studies have focused on the distributions of ARG and VF in giant panda-associated pathogens, captive–wild giant panda comparative analyses of ARGs and VFs, and the influence factors of ARG and VF distributions. However, a few studies have investigated the longitudinal dynamics of ARGs and VFs throughout the reintroduction process and a systematic analysis of possible causes affecting their distribution.

In this study, we employed metagenomic sequencing to investigate the dynamics of ARGs and VFs during giant panda reintroduction programs. Specifically, we aimed to (1) evaluate how geographic translocation and diet influence ARG and VF composition and (2) assess the potential for environmental transmission of ARG and VF, and provide actionable insights for optimizing reintroduction protocols and the safety of the public environment.

## 2. Materials and Methods

### 2.1. Sample Source

The data utilized in this study were derived from our previous work, and we reanalyzed these fecal metagenome data [25]. Fifteen fecal samples from giant pandas (*Ailuropoda melanoleuca*) at three distinct stages (semi-wild, release, and wild) were collected from the Xiaoxiangling Mountains between 2012 and 2016. Four fecal samples were collected during the semi-wild stage, four fecal samples during the release stage, and seven fecal samples during the wild stage. Samples from the semi-wild and release stages were obtained from three giant pandas translocated to the Xiaoxiangling Mountains (Zhangxiang, ZX; Taotao, TT; and Huajiao, HJ). The wild-stage fecal samples were collected from the Xiaoxiangling Mountains and one wild translocated individual (Luxin, LX). The fecal samples were aseptically collected from giant pandas with a GPS collar by the monitoring team. To maintain the integrity of the samples, fresh fecal samples of giant pandas were collected immediately, within 10 min after defecation. Using sterile disposable nitrile gloves, fresh fecal samples were carefully transferred into pre-labeled, DNA/RNA-free sterile collection bags with minimal environmental contact. The samples were kept frozen on dry ice after collection, immediately transported to the laboratory on dry ice, and stored at −80 °C until subsequent DNA extraction. The detailed collection dates and sample origins were recorded in Table S1.

### 2.2. DNA Extraction

The fecal samples from giant pandas were initially soaked in anhydrous ethanol for 24 h to stabilize the microbial DNA and inhibit degradation. Subsequently, the samples were subjected to repeated centrifugation (3000× *g*, 10 min, 4 °C) to enrich the precipitate for downstream genomic DNA extraction. The total DNA was extracted from the fecal samples using the Qiagen DNA Stool Kit (Qiagen, Hilden, Germany), according to the manufacturer’s protocol. The DNA quality was assessed by 1% agarose gel electrophoresis and quantified using the Qubit 4.0 Fluorometer (Thermo Fisher Scientific, Waltham, MA, USA) with the dsDNA HS Assay Kit (Thermo Fisher Scientific, Waltham, MA, USA), requiring minimum concentrations of 10 ng/µL and A260/A280 ratios between 1.8 and 2.0.

### 2.3. Metagenomic Sequencing

For the library preparation, 100 ng of high-molecular-weight DNA was mechanically fragmented to an optimal insert size of 450 bp using a Covaris M220 ultrasonicator (Covaris, Woburn, MA, USA). A paired-end library was constructed using the TruSeq™ DNA PCR-Free Library Preparation Kit (Illumina, San Diego, CA, USA), following the manufacturer’s protocols. Briefly, the sample DNA was ligated to Illumina-compatible Y-shaped adapters. Adapter-dimers and self-ligated fragments were removed through bead selection. Then, the library templates were enriched via a PCR cycle (initial denaturation at 98 °C for 45 s; cycling at 98 °C for 15 s, 60 °C for 30 s, and 72 °C for 30 s; final extension at 72 °C for 5 min). The resulting library was finally denatured with NaOH to generate single-stranded DNA for cluster generation. The prepared DNA libraries were hybridized with complementary oligonucleotides immobilized on the flow cell surface using the cBot TruSeq PE Cluster Kit v3 (Illumina, San Diego, CA, USA). Subsequent bridge amplification was performed under isothermal conditions to generate clusters. All samples were sequenced on the Illumina HiSeq 2500 platform with the pair-end 150 bp (PE150) mode by Shanghai Biozeron Biotechnology Co., Ltd. (Shanghai, China).

### 2.4. Data Analysis

Raw sequencing data underwent rigorous quality control and preprocessing prior to a downstream analysis. An initial sequence quality assessment was performed using FastQC (v0.12.1) [26]. The raw sequence reads were subsequently trimmed using Trimmomatic (v0.39) [27] to remove adaptor contaminants and low-quality reads with the following optimized parameters: filtering bases with a mass value of 20 or less at the tail of the reads; implementing sliding-window trimming (window size: 50 bp) starting from the 5’ end; truncating bases when the average quality score (Q) ≤ 20 within any window; and removing reads containing >2 ambiguous bases, adapter-contaminated reads, and reads with lengths < 50 bp. BWA-MEM (v0.7.17) [28] was used for alignment against the giant panda reference genome to remove host-derived sequences. The reads with removed host-genome contaminations and low-quality data were called clean reads and used for further analyses. Then, the clean reads were assembled into contigs using Megahit (v1.2.9) (minimum contig length: 500 bp) [29]. Salmon was used to calculate the coverage of contigs and remove contigs with less than 60% coverage [30]. The gene prediction in contigs was performed with Prodigal (v2.6.3) [31], obtaining gene files for each metagenome. A non-redundant gene catalog was constructed through clustering with CD-HIT (v4.8.1) at 95% sequence identity and 90% coverage thresholds [32]. We used Salmon to post back the clean reads to the non-redundant gene set and obtained the TPM (transcripts per million reads) abundance of these non-redundant gene profiles in each metagenome.

Using BLASTP (http://blast.ncbi.nlm.nih.gov/Blast.cgi) (accessed on 25 June 2025), we performed a sequence similarity search between the non-redundant gene set and the NR database, applying stringent thresholds (e-value ≤ 1 × 10^−5^, identity ≥ 55%, and score ≥ 60). Based on the alignment results, we utilized a custom Perl script to integrate with NR’s taxonomic classification data and annotate the species of the non-redundant gene set. Then, according to the NR species annotation results and gene abundance tables, we used a custom Perl script to obtain TPM abundance for each species at the taxonomic level. High-quality sequencing reads were aligned against the Structured Antibiotic Resistance Gene Database (SARG v3.2) [33] using BLASTP [34] with stringent criteria (e-value ≤ 1 × 10^−7^, sequence identity ≥ 60%). Based on the comparative analysis results, ARGs were systematically annotated against the SARG v3.2 through a custom Perl script. Using the annotated ARGs and their corresponding abundance profiles, we generated ARG type and subtype abundance tables (TPM abundance). For the virulence factors, sequences were aligned against the Virulence Factor Database (VFDB) by applying comparable thresholds (e-value ≤ 1 × 10^−5^, identity ≥ 60%). The obtained virulence gene annotation information was then combined with the gene abundance to construct the virulence gene abundance table (TPM abundance). The VFs were systematically classified according to functional categories and name. Non-metric multidimensional scaling (NMDS) based on the Bray–Curtis distance [35] was performed to identify potentially distinct clusters among the different stages of giant pandas. A comparative analysis of ARGs and VFs based on the Bray–Curtis distance was conducted to compare the magnitude of the differences across different stages. To visualize taxonomic contributions to ARG and VF profiles, we used a custom Perl script to create the relationship between ARGs/VFs and taxonomy based on the annotations and abundance information of ARG types, ARG subtypes, VF function categories, VF names, and taxonomy summary. According to these statistics, a configuration file was generated and imported into Circos [36] for graphing.

## 3. Results

### 3.1. The Distributions of ARG in Released Giant Pandas Exhibited Similarity to Wild Giant Pandas

**Figure 1:**
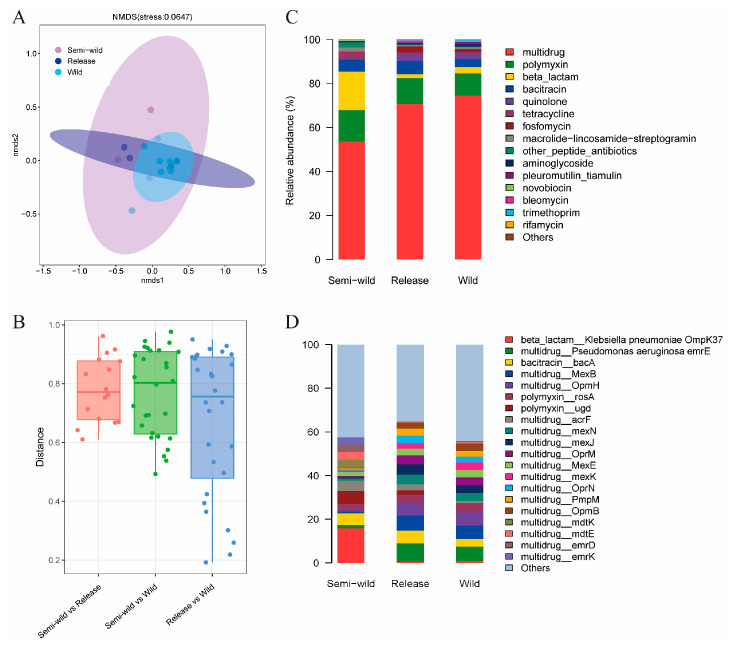
The changes in gut microbial antibiotic resistance genes (ARGs) across giant pandas. (**A**) Non-metric multidimensional scaling (NMDS) based on Bray–Curtis distance of ARGs showing distinct clustering patterns among semi-wild, released, and wild giant pandas. (**B**) Boxplot showing Bray–Curtis distances to compare the ARG differences between different stages. (**C**,**D**) Relative abundance of dominant ARG types and subtypes in different stages of giant pandas.

Non-metric multidimensional scaling (NMDS) based on Bray–Curtis distance of ARGs in the gut microbiomes of giant pandas revealed distinct clustering patterns across different stages: semi-wild, release, and wild. The released giant pandas’ ARG profiles were similar to the wild stage (Figure 1A). A comparative analysis between the groups based on Bray–Curtis distance further demonstrated that pronounced disparity occurred between semi-wild and wild populations, whereas released giant pandas showed reduced difference of ARG to wild stages (Figure 1B). An ARG composition analysis identified multidrug, polymyxin, and β-lactam resistance genes as the predominant types in semi-wild giant pandas. Released and wild giant pandas shared similar ARG compositions, both dominated by multidrug and polymyxin resistance genes. Compared to semi-wild individuals, released and wild giant pandas exhibited an increased relative abundance of multidrug resistance genes and a reduction in polymyxin and β-lactam resistance genes (Figure 1C). In addition, an analysis of ARG subtypes further revealed rich ARGs in giant pandas and variations in the distribution patterns across different stages. Notably, the semi-wild stage exhibited a high abundance of the *Klebsiella pneumoniae* ompk37-type resistance gene. The ARG profiles between the released and wild giant pandas showed greater similarity, showing high abundance of *Pseudomonas aeruginosa* emrE, bacA, MexB, and OmhH (Figure 1D).

### 3.2. The Distributions of VF in Released Giant Pandas Exhibited Similarity to Wild Giant Pandas

**Figure 2:**
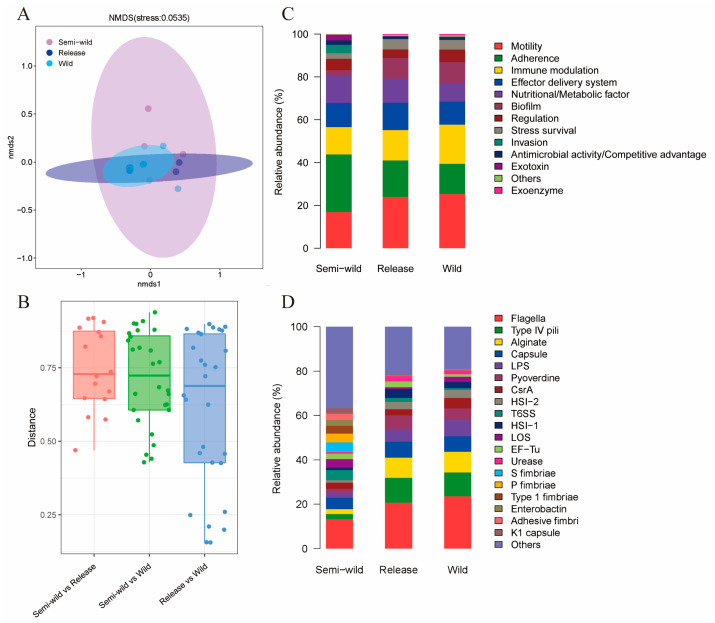
The changes in gut microbial virulence factors (VFs) across giant pandas. (**A**) Non-metric multidimensional scaling (NMDS) based on Bray–Curtis distance of VFs showing distinct clustering patterns among semi-wild, released, and wild giant pandas. (**B**) Boxplot showing Bray–Curtis distance to compare the VF differences between different stages. (**C**,**D**) Relative abundance of dominant VFs classified by functional categories and names in different stages of giant pandas.

An NMDS analysis based on Bray–Curtis distance of VFs in giant pandas’ intestinal microbiomes revealed distinct clustering patterns among semi-wild, released, and wild populations. Notably, the VF distributions in released giant pandas exhibited closer resemblance to wild individuals (Figure 2A). A comparative analysis further demonstrated that the differences in VF in giant pandas gradually decreased as the release proceeded, with the smallest dissimilarity observed between released and wild giant pandas (Figure 2B). In addition, the distribution patterns of VF in the released and wild stages were similar. The VFs in the three stages were mainly based on the functional categories of motility, adherence, immune modulation, effector delivery systems, and nutritional/metabolic factors. However, the differences were that released and wild giant pandas exhibited an increased relative abundance of motility-associated VFs, alongside reduced proportions of VFs associated with adherence and nutritional/metabolic factors compared to semi-wild individuals (Figure 2C). Subsequently, we analyzed the abundance of VF names in different stages, suggesting rich types of VF at all stages of the reintroduction program in giant pandas. The semi-wild stage was enriched in flagella-associated virus factors, while the released and wild giant pandas were mainly dominated by the virus factors of flagella, type IV pili, alginate, capsule, and LPS (Figure 2D).

### 3.3. ARGs and VFs with Increased Abundance in Released and Wild Giant Pandas Were Mainly Found in Pseudomonas

**Figure 3:**
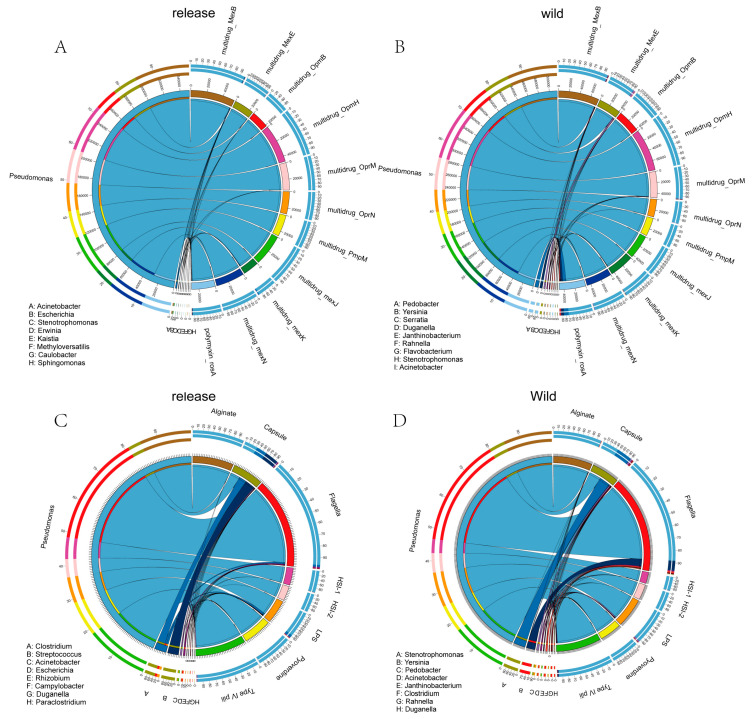
The distributions of ARG and VF in released and wild giant pandas. (**A**,**B**) The distributions of ARG subtypes with increased abundance in released and wild giant pandas compared to the semi-wild stage. (**C**,**D**) The distributions of VF categorized by names with increased abundance in released and wild giant pandas compared to the semi-wild stage.

We analyzed the microbial distribution of increased abundance in ARG subtypes and VF names in both released and wild giant pandas compared to semi-wild stages. In both released and wild giant pandas, ARGs were mainly found in *Pseudomonas*, with minor contributions from other bacterial genera. Similarly, the VFs were also mainly identified in *Pseudomonas*. Notably, VFs associated with capsule and flagella function exhibited distinct distribution between the two groups. In released giant pandas, the VFs associated with capsule function were primarily found in *Pseudomonas*, *Clostridium*, and *Streptococcus*, whereas in wild giant pandas, *Pseudomonas* and *Stenotrophomonas* were the predominant bacterial distribution. For flagella-associated virulence factors, released giant pandas were mainly found in *Pseudomonas*, while in the wild group, they were additionally identified in *Yersinia* (Figure 3A–D).

## 4. Discussion

### 4.1. Characterization of Intestinal Microbial Resistance and Virulence Factors at Different Stages of Giant Pandas: Potential Risks for Wild Release

Antibiotics are extensively employed for the prevention and treatment of infectious diseases in giant pandas, which may contribute to the emergence of multidrug-resistant bacterial strains. Our findings demonstrated that ARGs in giant pandas across different stages were predominantly multidrug resistance genes, which is consistent with previous studies [37]. Notably, semi-wild giant pandas exhibited an elevated abundance of β-lactamase resistance genes. β-lactamase is one of the veterinary antibiotics frequently used for captive giant pandas [38]. This observation likely reflected the accumulation of resistance genes resulting from antibiotic overuse in a captive environment. Furthermore, all stages of giant pandas showed a high prevalence of polymyxin resistance genes. Polymyxins represent the last-resort antibiotics against MDR Gram-negative bacterial infections [39]. The widespread detection of polymyxin resistance highlighted the severity of antimicrobial resistance in giant pandas, underscoring the urgent need for improved antibiotic stewardship in both captive and wild management practices. The presence of ARGs was detrimental to the treatment of infections in giant pandas, and resistant genes may be transmitted to the environment through wild release, posing a serious threat to public health safety.

In terms of VFs, our analysis revealed similar VFs between released and wild giant pandas, with flagella, type IV pili, capsule, alginate, and lipopolysaccharide (LPS) representing the predominant VFs. In contrast, semi-wild giant pandas contained more abundant VFs, mainly dominated by flagella. Studies have shown that flagella and type IV pili can enhance microbial motility, adhesion, and invasion capabilities by activating flagellar assembly pathways and bacterial chemotaxis systems [40]. Capsule and alginate can facilitate biofilm formation and help pathogens to carry out immune escape and anti-phagocytosis [3,41,42]. LPS can mediate host–pathogen interactions by triggering pattern recognition receptors, subsequently inducing inflammatory responses and compromising host immunity [43]. These findings have underscored how VFs promote pathogenic colonization while undermining host health, posing significant conservation challenges for giant panda management.

### 4.2. Geography and Diet May Be Key Drivers of the Distribution Patterns of ARG and VF in Giant Pandas’ Different Stages

ARGs and VFs in giant pandas can be affected differently by geography. Hu et al. found that the composition of ARG exhibits notable variations between captive and wild giant pandas, and the distinct differences were observed in the Qinling population compared to others, probably due to the geographic isolation from other wild populations [22]. The VF distribution also has been observed to show notable differences between captive and wild giant panda populations [21,44]. In addition, it has been found that there are different distribution patterns of ARG in crested ibis (*Nipponia nippon*) across three geographically distinct environments—captive, feral, and wild populations—with high similarity observed between fecal ARG profiles and those detected in their respective habitat [45]. Environmental factors play a crucial role in shaping the ARG profiles of the giant pandas’ gut microbiomes [46]. ARGs and VFs can be horizontally transferred via MGEs between environmental media (air, water, soil, plants) and host organisms [47,48,49], enabling different ARGs and VFs to be shared in gut and habitat environments. In this study, we examined the dynamics of ARGs and VFs in semi-wild, released, and wild giant pandas. Our findings revealed that the ARGs and VFs of released giant pandas gradually converge toward wild giant pandas. This shift is likely driven by the environmental transmission of ARGs and VFs in the same geographic environment.

Diet also plays an important role in the distributions of ARG and VF in the gut microbiomes. A study on canine and feline gut resistomes has demonstrated that elevated dietary protein and reduced carbohydrate intake correlate with increased ARG diversity [50]. Similarly, ARG profiles were found to have significant differences in giant pandas consuming different bamboo species (*Chimonobambusa szechuanensis* and *Bashania fangiana*) [51]. Furthermore, higher dietary flavonoid intake in giant pandas is considered to be associated with reduced gut microbial diversity and diminished abundance of VFs [44]. Diet-mediated bacterial remodeling may serve as the main target of most ARGs in the gut [52]. Bamboo-associated microbiome ingested with the diet can modify the gut microbial composition, thereby altering the antibiotic resistance profile [53]. Our findings revealed that, compared to semi-wild giant pandas, ARGs and VFs elevated in released and wild giant pandas were mainly identified in *Pseudomonas*. We speculated that bamboo consumption may influence the composition of giant pandas’ gut microbiomes (high proportion of *Pseudomonas*) [16,25,54], ultimately influencing the distributions of ARG and VF in host bacteria.

### 4.3. Pseudomonas May Serve as a Critical Reservoir of Antibiotic Resistance and Virulence Factors in Giant Pandas: Implications for Public Health

Among the genus of *Pseudomonas*, *Pseudomonas aeruginosa* represents the most common opportunistic pathogen, capable of causing severe infections in humans, including cystic fibrosis, urinary tract infections, meningitis, and *Pseudomonas* bacteremia [55,56,57]. *Pseudomonas* has demonstrated resistance to multiple classes of antibiotics, including aminoglycosides, quinolones, and β-lactams [58]. The escalating prevalence of antibiotic resistance has led to the emergence of clinically challenging strains, such as carbapenem-resistant *Pseudomonas aeruginosa* (CRPA) [59], multidrug-resistant *Pseudomonas aeruginosa* (MDRPA) [60], and carbapenem resistance in difficult-to-treat *Pseudomonas aeruginosa* (DTR-PA) [61]. These resistant phenotypes significantly complicate therapeutic interventions, posing a formidable challenge in clinical settings. In our study, we found that the elevated ARGs and VFs in released and wild giant pandas were mainly found in *Pseudomonas*, demonstrating that *Pseudomonas* may serve as a rich reservoir of ARGs and VFs in giant pandas. The presence of rich ARGs and VFs in *Pseudomonas* from giant pandas underscored the potential for transmission to the environment or humans, particularly in areas where human activities overlap with wildlife habitats, causing serious clinical infections. Furthermore, the horizontal transfer of ARGs and VFs within *Pseudomonas* may accelerate the emergence of clinically serious drug-resistant strains. Meanwhile, it is also important to be vigilant of the serious dangers of the emergence of *Pseudomonas* strains with simultaneously equipped drug resistance and high virulence.

Our study elucidated the dynamic changes in ARGs and VFs in the gut microbiomes of giant pandas across different reintroduction stages while discussing the impacts of geographical and dietary factors on ARGs and VFs, providing critical theoretical support for giant pandas’ conservation management. We proposed to conduct the longitudinal monitoring of ARGs and VFs in giant pandas to assess individual health risks and mitigate environmental dissemination of multidrug-resistant bacteria and associated genetic elements. Furthermore, the surveillance of ARGs and VFs can guide captive antibiotic protocols. Our study provided novel insights into the ARGs and VFs of the giant panda gut microbiomes, although several limitations should be acknowledged. Firstly, the sample size in our study was relatively small, future studies should expand to larger, geographically diverse populations to comprehensively assess the distribution patterns of ARG and VF across different habitats. Such efforts will facilitate targeted surveillance and evidence-based management of antimicrobial resistance in this endangered species. Additionally, in future studies, we plan to characterize MGEs in the giant pandas’ gut microbiomes and evaluate their roles in ARG and VF dissemination using metagenomic assembly and network analyses.

## 5. Conclusions

Our study revealed distinct differences in the compositions of ARG and VF between semi-wild giant pandas and released or wild giant pandas, while the released and wild populations demonstrated similarities. These likely resulted from the transmission of ARGs and VFs between the host and the environment. In addition, we found that the ARGs and VFs increased in released and wild giant pandas were mainly found in *Pseudomonas*. We hypothesized that bamboo consumption may modulate the gut microbial composition in giant pandas, consequently influencing the distribution patterns of ARG and VF within the intestinal microbiomes. In conclusion, this study can provide critical insights for optimizing giant panda conservation strategies while addressing emerging challenges in ecosystem health and public safety. Our study advocates that the monitoring of ARGs and VFs in giant pandas can be considered in reintroduction programs to enable individualized health risk assessments and prevent the spread of high-risk ARGs and VFs into the environment through transmission. Furthermore, it can support the rational use of antibiotics for giant pandas in captivity according to the actual local drug resistance situation.

## Data Availability

The metagenomic data presented in this study can be found in figshare at https://doi.org/10.6084/m9.figshare.6303713.

## Supplementary Materials

The following supporting information can be downloaded at https://www.mdpi.com/article/10.3390/microorganisms13071616/s1, Table S1: The sample information in this study.
